# Toward the generation of pure coral genomes with experimental and bioinformatic improvements

**DOI:** 10.1016/j.xinn.2024.100643

**Published:** 2024-05-20

**Authors:** Yisi Hu, Zhiwei Zhang, Shuyan Sun, Youfang Sun, Hui Huang, Wenliang Zhou, Fuwen Wei

**Affiliations:** 1 Center for Evolution and Conservation Biology, Southern Marine Science and Engineering Guangdong Laboratory (Guangzhou), Guangzhou 511458, China; 2 Jiangxi Provincial Key Laboratory of Conservation Biology, College of Forestry, Jiangxi Agricultural University, Nanchang 330029, China; 3 Key Laboratory of Animal Ecology and Conservation Biology, Institute of Zoology, Chinese Academy of Sciences, Beijing 100101, China; 4 Key Laboratory of Tropical Marine Bio-resources and Ecology, South China Sea Institute of Oceanology, Chinese Academy of Sciences, Guangzhou 510301, China

## Abstract

Stony corals, the primary architects of coral reef ecosystems, are largely underrepresented in omics studies despite their importance. The presence of endosymbiotic Symbiodiniaceae algae complicates the extraction of pure coral DNA, posing a challenge for genomic research. Here, we devised a comprehensive methodological framework that incorporates various experimental treatments to achieve 99% purity in coral DNA extraction and a robust bioinformatics pipeline to guarantee the assembly of high-quality, contamination-free coral genomes. Validation of our framework using *Acropora millepora* samples demonstrated its efficacy and superiority in obtaining high-quality pure coral genomes using easily accessible adult colony. This integrated framework serves as a critical foundation for large-scale genome-enabled research on stony corals, providing insight into coral evolution and conservation.

## Main text

The advancement of genomics technology has revolutionized research on diverse taxa, significantly improving our understanding of species biology and evolution. Genome sequencing is the initial step required for comprehensive representation of a species’ genome and provides reference data for further analyses. The genomes of numerous marine species remain unexamined, because either their importance is overlooked or there are significant challenges associated with acquiring biological samples.^1^ Stony corals are foundational reef builders that house one-third of marine biodiversity and act as an important carbon sink, making them essential for marine conservation and sustainable development.^2^^,^^3^ They have a symbiotic relationship with single-celled algae (Symbiodiniaceae) that reside within coral symbiotic cells. These algae supply corals with energy through photosynthesis and underpin the growth and development of coral reefs.

However, these endosymbiotic partners pose a challenge to genomic research because their DNA is often coextracted with coral DNA, leading to contamination and assembly errors. Additionally, the presence of microorganisms and invertebrates within the porous structure of coral skeletons further complicates the extraction of the DNA composition of stony coral samples. Failure to detect nontarget sequences from associated organisms in the genome assembly and the lack of reliable coral genomic resources may result in spurious interpretation of species evolutionary history and metabolic potential. This can obstruct our understanding of coral adaptation, evolution, and response to environmental changes, leading to missed opportunities for biotechnology applications and targeted conservation strategies.^4^

To date, whole-genome sequencing has been performed for only 2.5% of nearly 1,700 stony coral species. Previous studies have mostly used Symbiodiniaceae-free gamete cells collected during coral spawning to avoid potential contamination.^5^^,^^6^ However, spawning activity only occurs a few nights per year, and this method is not applicable for species with vertical symbiont transmission (in which endosymbiotic algae are transmitted from the last generation through gametes) or for brooding species (in which fertilization and early coral development occur internally, and only Symbiodiniaceae-hosting larvae are released). Thus, considerable taxonomic bias exists in coral genomic research, with *Acropora* species accounting for half of the sequenced stony coral genomes. The challenges posed by symbiont contamination to genome sequencing of corals can be observed in a recent study of the reef-building blue coral (*Heliopora coerulea*) where adult coral fragments were sampled.^7^ The initial assembly with algal and bacterial contamination was 3 times larger than the final assembly after filtering (1.3G vs. 428M).^7^ The filtering process may have also compromised genome integrity. Clearly, gathering uncontaminated coral samples for sequencing is essential under current methodologies, and new bioinformatics pipelines are required to address contamination issues.

Here, we present several experimental methods for obtaining clean coral DNA samples from adult tissues for genome sequencing of various stony coral species. We describe a bioinformatics framework to mitigate the assembly errors caused by contamination. This methodological framework for generating pure coral genomes is a considerable advance on existing methodologies. It offers convenient and low-cost methods that overcome the challenges of acquiring suitable samples with limited resources from diverse species and removes the risk of equivocal outcomes from contaminated samples that attend current practices. This new methodology will advance coral genomics on a large scale and ultimately contribute to the conservation and sustainable management of coral reefs.

## Obtaining clean coral samples

### Experimentally removing symbiotic algae from adult coral tissues

**Figure 1:**
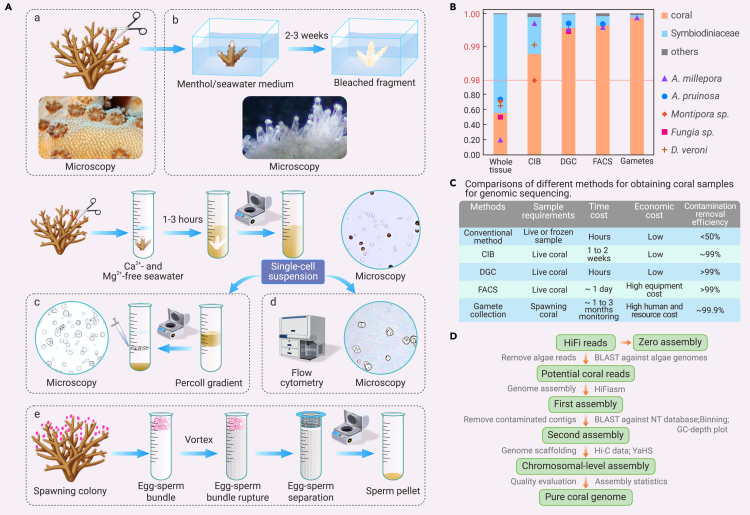
The integrated experimental and bioinformatic framework to generate pure coral genomes (A) Illustration of different methods used to obtain coral samples for genome sequencing, including (a) conventional method using whole tissue, (b) chemical-induced bleaching (CIB) method that generates aposymbiotic coral, (c) density gradient centrifugation (DGC) method that separates asymbiotic coral cells from cell suspension using a Percoll gradient, (d) fluorescence-activated cell sorting (FACS) method that isolates nonfluorescent coral cells from cell suspensions using flow cytometry, and (e) gamete collection method that collects egg-sperm bundles from a spawning colony and uses sperm for DNA extraction. Microscopic examination of the coral cells obtained through CIB, DGC, and FACS methods revealed the absence of Symbiodiniaceae. (B) Relative proportions of corals, Symbiodiniaceae, and other eukaryotes in the coral DNA samples from different methods in five species (represented by different symbols). The y axis (proportional representation) is magnified from 0.98 to 1.00 for better visualization of the differences between samples. (C) Comparison of different methods for obtaining coral samples for genomic sequencing. (D) Bioinformatics pipeline for removing contamination and improving genome assembly.

To obtain clean coral samples without Symbiodiniaceae contamination, we examined three experimental treatments and compared them with the conventional method and the gamete collection method. We tested five species from both the Complexa and Robusta clades of Scleractinia (*A. millepora*, *A. pruinosa*, *Dipsastraea veroni*, *Montipora* sp., and *Fungia* sp.) (Figure 1A). The first treatment, chemical-induced bleaching (CIB), involves the use of menthol stimulation to deplete Symbiodiniaceae from coral hosts and generate aposymbiotic live corals within a 2-week time frame^8^ (Figure 1Ab). The second (density gradient centrifugation, DGC) and third (fluorescence-activated cell sorting, FACS) treatments involve isolating asymbiotic coral cells (such as gland cells and supporting cells) from alga-hosting cells and algal cells from live corals; the former are used for coral DNA extraction. Both methods start by preparing cell suspensions by detaching coenosarc tissues from the coral skeleton using calcium- and magnesium-free artificial seawater and disassociating them into individual cells. The DGC method employs Percoll medium to isolate different cell types based on differences in density. Asymbiotic coral cells with lower densities than alga-hosting cells and algal cells are specifically collected via centrifugation (Figure 1Ac). The FACS method uses the autofluorescence property of Symbiodiniaceae to sort the cell suspension into nonfluorescent coral cells and other alga-containing cells or algal cells through flow cytometry (Figure 1Ad). In comparison, the conventional method extracts DNA directly from whole coral tissues, which unavoidably contains undesired contaminants from endosymbiotic algae and other associated organisms (Figure 1Aa). The gamete collection method is usually used on wild coral colonies under routine monitoring. Gamete bundles naturally devoid of Symbiodiniaceae are collected during coral spawning in the breeding season and used for coral DNA extraction (Figure 1Ae). The detailed methodology of the experiments above is available at https://doi.org/10.6084/m9.figshare.24947382.

### Determination of symbiont removal efficiency based on metabarcoding

To assess the efficacy of our experimental treatments in eliminating symbiotic algae, we conducted metabarcoding. We used a eukaryotic conserved 18S rRNA sequence on DNA samples from the five species to determine the species composition within these samples (Figure 1B). Samples from the same species were grouped into matched pairs (treatment vs. control) for comparison. Compared to the conventional method, which typically yielded 55.3% ± 19.5% (standard deviation) coral DNA, 44.4% ± 19.6% Symbiodiniaceae DNA, and 0.03% ± 0.05% DNA from other species, all three experimental treatments we used consistently produced at least 98.8% coral DNA, with an average improvement of 50.6% ± 23.6% in coral DNA content between matched pairs. Among the three treatments, CIB presented the lowest coral DNA content with the highest variation (98.80% ± 0.08%), while DGC and FACS demonstrated similarly high levels of coral DNA (99.56% vs. 99.63%), with minimal variation. These values were all comparable to the coral DNA content in the gamete sample (99.9%). Apart from Symbiodiniaceae, Apicomplexa, a taxon of parasitic single-cell organisms widely found in corals, is the most frequently detected contaminating eukaryote in coral samples, with a relative abundance of 0.038% ± 0.052%. Other contaminants included single-celled green algae (such as *Ostreobium* spp.) and annelids that colonized the coral skeleton.

### Comparing the experimental treatments

Based on a combined assessment of sample requirement, time cost, economic cost, and contamination removal efficiency (Figure 1C), we find that DGC is the most cost-effective option. The DGC method requires the addition of only a few steps to the conventional method yet is very efficient in removing symbiont contamination. The CIB method is comparatively time-consuming, but it significantly reduces Symbiodiniaceae across a wide range of coral taxa.^8^^,^^9^ In contrast, despite superior performance in removing algae, the FACS method requires expensive equipment and multiple runs to collect adequate cells (∼10^7^), making it a less economical choice for genomic sequencing. Nonetheless, all these methods offer great advantages over the conventional method by providing greater flexibility in their application as well as robust results for a wide range of stony coral species. In particular, these new methods are not limited by the need to collect gametes. Using samples of the easily accessible adult colony paves the way toward increasing the number of coral species whose genome can be sequenced.

## Improving coral genome assembly

In addition to ensuring the single-taxon origin of genomes from decontaminated coral samples, we also devised a bioinformatics pipeline to improve the genome assembly. We conducted PacBio HiFi sequencing on three DNA samples (CIB, DGC, and gamete) of *A. millepora* to assess the performance of our pipeline in assembling high-quality genomes for the same species. In brief, the procedure involved the following: (1) generating the zero assemblies as benchmarks with all sequencing reads; (2) removing HiFi reads aligned with published Symbiodiniaceae genomes and generating the first assemblies with the remaining reads; (3) identifying contaminated contigs by both taxonomy-annotated sequence similarity matches and reference-free contig clustering; (4) combining results from the previous step to remove contaminated sequences and to generate the second assemblies; (5) performing genome scaffolding using Hi-C data; and (6) evaluating the quality of the final assemblies (Figure 1D).

In the second step, consistent with metabarcoding analyses, only 0.1% of the reads were recognized as Symbiodiniaceae-derived reads, and these were subsequently excluded. In the third and fourth step, we combined sequence similarity searches (reference based) and contig clustering based on the base composition and read coverage (reference free) to detect potential contaminants from other taxa. No contaminated sequence was found for the CIB sample. 1.73-Mb sequences of *Vibrio* bacteria were identified by database searches and genomic binning for the DGC sample and verified by GC-depth plot visualization. Three contigs with a total length of 240 kb were identified as the mitochondrial, chloroplastic, and nuclear rRNA sequences of a Streptophyta plant, most likely from anthropogenic contamination. We also identified one contig (22 kb) potentially from Apicomplexa in the first assembly of the gamete sample, which aligned with the metabarcoding results. Based on the second assemblies of the three samples, we conducted scaffolding using Hi-C data to generate the final chromosome-level genome assemblies. The three final assemblies exhibited high quality in terms of continuity and integrity, with contig N50 values exceeding 14 Mb, scaffold N50 values exceeding 30 Mb, and BUSCO completeness for genome exceeding 93.2% (metazoa_odb10). Together, these results indicate the excellent efficacy of the experimental treatments in yielding pure coral DNA and the advantages of our bioinformatic pipeline for the assembly of high-quality pure coral genomes.

When applying our bioinformatics framework to a published *A. millepora* genome using both gametes and adult tissues,^10^ we also found genomic regions that were more likely to have originated from Symbiodiniaceae than from corals (e.g., sequence NW_025322990.1). Therefore, unintended errors may occur when using the public data in the NCBI database. We recommend that future studies implement more stringent identification of contamination using the framework we propose, both in genome assembly and analyses of public data.

## Prospects

Amid escalating rates of coral loss, our integrated framework offers a valuable initial step in overcoming current limitations to obtaining reliable representations of more coral genomes on a large scale. This advancement holds significant promise for facilitating more coral genomic sequencing projects, potentially enabling the assembly of telomere-to-telomere genomes for species revival in the event that they are locally extirpated. Unraveling coral genomic architecture is also essential for understanding coral evolutionary history, population dynamics, and adaptation across different environments, which is indispensable for effective species conservation. In addition, our innovative methods hold the potential to segregate holobiont data for in-depth omics analyses, shedding light on the separate examination of each player’s functions and their crosstalk during coevolution. These research insights are key to bolstering the long-term conservation of threatened coral reefs worldwide.
